# A closer look at disparities in earnings between white and minoritized dentists

**DOI:** 10.1111/1475-6773.14095

**Published:** 2022-11-14

**Authors:** Kamyar Nasseh, Bianca K. Frogner, Marko Vujicic

**Affiliations:** ^1^ Health Economist, Health Policy Institute American Dental Association Chicago Illinois USA; ^2^ Department of Family Medicine University of Washington School of Medicine Seattle Washington USA; ^3^ Chief Economist & Vice President Health Policy Institute, American Dental Association Chicago Illinois USA

**Keywords:** dentist earnings, Oaxaca decomposition, racial disparities

## Abstract

**Objective:**

To examine the factors that account for differences in dentist earnings between White and minoritized dentists.

**Data Sources:**

We used data from the American Dental Association's Survey of dental practice, which includes information on 2001–2018 dentist net income, practice ZIP code, patient mix between private and public insurance, and dentist gender, age, and year of dental school graduation. We merged the data on dentist race and ethnicity and school of graduation from the American Dental Association masterfile. Based on practice ZIP code, we also merged the data on local area racial and ethnic composition from the American Community Survey.

**Study Design:**

We used a linear Blinder‐Oaxaca decomposition to assess observable characteristics that explain the gap in earnings between White and minoritized dentists. To assess differences in earnings between White and minoritized dentists at different points of the income distribution, we used a re‐centered influence function and estimated an unconditional quantile Blinder‐Oaxaca decomposition.

**Data Extraction Methods:**

We extracted data for 22,086 dentists ages 25–85 who worked at least 8 weeks per year and 20 hours per week.

**Principal Findings:**

Observable characteristics accounted for 58% of the earnings gap between White and Asian dentists, 55% of the gap between White and Hispanic dentists, and 31% of the gap between White and Black dentists. The gap in earnings between White and Asian dentists narrowed at higher quantiles of the income distribution.

**Conclusions:**

Compared to other minoritized dentists, Black dentists have the largest earnings disparities relative to White dentists. While the level of the explained component of the disparity for Black dentists is comparable to the explained part of the disparities for other minoritized dentists, the excess percentage of the unexplained component for Black dentists accounts for the additional amount of disparity that Black dentists experienced. Persistent income disparities could discourage minoritized dentists from entering the profession.

**What is known on this topic**

- There are observable wage gaps between racial and ethnic groups in many health care professions, but these gaps are not fully explained by education or other demographic characteristics.
- Wage gaps between male and female dentists exist after controlling for certain practice characteristics. Similar analysis has not been done for dentist race and ethnicity.

**What this study adds**

- Previous research has examined the gap in earnings between White and minoritized medical providers, but little research has examined the gap in earnings between White and minoritized dentists.
- Despite controlling for numerous dentist and local area characteristics, we find that most of the gap in earnings between White and Black dentists remains unexplained.
- Unlike the gap in earnings between male and female dentists, which has narrowed in recent years, the gap in earnings between White and minoritized dentists has remained constant.

## 1 INTRODUCTION

The gap in dental care utilization between White and racial and ethnic minoritized populations in the United States has narrowed in recent years, particularly for children.^1^ A contributing factor may be the diversification of the dental workforce; studies have found that racial and ethnic concordance between providers and patients reduces health care disparities.^2^, ^3^ As of 2020, 70% of dentists are White, while White individuals constitute about 58% of the US population.^4^ Increasing diversity in the dentist workforce is largely due to an increasing percentage of Asian dentists.^5^ A concern is that existing and potentially persistent wage disparities may discourage minoritized populations from entering the dental field.^6^

Dentist participation in Medicaid and the Children's Health Insurance Program (CHIP) may contribute to wage disparities by race and ethnicity, given the higher participation of Black and Hispanic dentists compared to White, non‐Hispanic dentists in these insurance programs.^7^ Medicaid is known to be a less generous payer than private health insurance, possibly discouraging dentist participation.^8^, ^9^ If White dentists continue to lag in their participation in Medicaid programs relative to minoritized dentists, and if minoritized dentists leave or do not enter the profession due to lower earnings, this could adversely affect the diversity of dental providers. Supporting the diversity of the dental workforce is necessary to continue gains over the last several years in dental care use among Medicaid‐enrolled children, who are more likely to be from minoritized populations compared to those enrolled in private health insurance.^10^

Frogner and Schwartz (2021) examined the gap in earnings between White and minoritized physicians and other health care workers. Wage gaps persisted between racial and ethnic groups in many health care professions, but these gaps were not fully explained by education or other demographic characteristics. However, the study did not examine dentists separately.^11^ Also, the proportion of minorities in dentistry has been historically lower than the proportion of minorities in other health care professions.^4^, ^11^

In this study, we used multiple years of a nationally representative dental practice survey to identify how wage disparities by race and ethnicity have changed over time. We focused on the extent to which Medicaid participation may be contributing to wage disparities by employing a Blinder and Oaxaca decomposition methodology, a common approach to identifying contributors to wage gaps in the labor economics literature. We also examined how other community types, such as rural areas, dental health professional shortage areas, and historically marginalized communities, correlate with dentist earnings. We employed a re‐centered influence function (RIF) to examine differences in earnings at different points of the income distribution. This is the first robust analysis of dentist wage gaps. Our findings can guide policy makers on targeted investments to improve wage parity by race and ethnicity, thus increasing diversity.

## 2 METHODS

### 2.1 Data sources and sample

From the 2002–2019 American Dental Association (ADA) Survey of Dental Practice (SDP), we pooled 18 years of dental practice data to form a repeated cross‐section of dentists over time. Each year of SDP data consists of individual dentist‐level responses on practice characteristics from the previous year. Hence, our sample covers data from 2001 to 2018. The pooled SDP response rate over the sample period was 22.2%. The SDP, which has been used extensively in previous research,^12^, ^13^, ^14^, ^15^ covers responses from dentists from all 50 states and the District of Columbia and is a representative cross‐section of professionally active dentists in the United States. This survey includes information on staffing, dentist specialty, hours worked per week, weeks worked per year, practice size, ZIP code of primary practice, and dentist demographics such as gender, age, and year of graduation from dental school. The SDP also ascertains the percentage of a dentist's patient panel that is privately or publicly insured. Since there is no dental benefit in Medicare, the publically insured portion of a dentist's patient panel primarily represents individuals with Medicaid or CHIP benefits. Many Medicare Advantage plans have dental benefits, but these are administered by private insurance plans.^16^ From the ADA masterfile of professionally active dentists, we merged in information on dental school of graduation and dentist race and ethnicity. We restricted our analytic sample to dentists ages 25 through 85.

Our main dependent variable of interest is logged inflation‐adjusted hourly net income. Annual dentist net income, as asked of SDP respondents, is defined as income after expenses and business taxes, and captures earnings from salary, commissions, bonuses, and dividends. Following data restrictions used by other studies when comparing wages between various subgroups,^17^ we restricted our sample to dentists who worked at least 8 weeks per year and 20 h per week and earned at least $5.10 per hour in dollars in 2018. Previous research analyzing wage differences across race and ethnicity similarly imposed a wage restriction of $3.10 per hour in dollars in 2000, which equates to $5.10 per hour in 2018.^17^ We accounted for inflation over time by converting dentist net income to dollars in 2018 using the all‐items consumer price index.^18^ Based on hours worked per week and the number of weeks worked per year, we calculated the number of hours worked per year for each dentist. Using hours worked per year, we then computed inflation‐adjusted hourly net income. To mitigate the potential effect of outliers, we logged inflation‐adjusted hourly net income.

Our primary independent variable was the dentist's race and ethnicity. We first categorized dentists as Hispanic versus non‐Hispanic. Among non‐Hispanic dentists, we then categorized by race: White, Black, Asian, and other race (including American Indian, Alaska Native, and multiracial dentists). These categories are mutually exclusive. Minoritized dentists refer to those from Hispanic, Black, Asian, and populations of other races.

Using data from the 2015–2019 American Community Survey (ACS), we determined whether a dentist practiced in a ZIP code where non‐Hispanic White individuals were less than 50% of the racial distribution.^19^ We also categorized practice location based on the rural–urban commuting area (RUCA) classifications, including large rural, small rural, isolated and urban.^20^ We collapsed large rural, small rural, and isolated areas into a general rural ZIP code category. We also categorized dentists according to the Bureau of Economic Analysis (BEA) regions: New England, Mideast Atlantic, Great Lakes, Plains, Southeast, Southwest, and Rocky Mountain/Far West. The BEA groups the states based on measures of localized economic activity and growth.^21^ By year and census region, we also merged in a regional consumer price index (CPI)^22^ to account for differences in the cost of living across the country, which could affect disparities in dentist earnings.

The pooled sample from the 2002–2019 SDP included 43,879 observations. Our final analytic sample included 22,086 observations after imposing wage restrictions and dropping missing observations on inflation‐adjusted hourly dentist net income, race and ethnicity, patient mix (percent insured by Medicaid), age, experience (defined as the number of years since graduation from dental school in five‐year increments), gender, specialty (general practice [GP] or specialist [pediatric dentist, endodontist, periodontist, prosthodontist, orthodontist, oral surgeon, or other specialists]), and primary practice ZIP code. We included a binary foreign dental school indicator variable since previous research concluded that country of origin and location of study can explain levels of workers' earnings.^23^ To account for differential nonresponse across various subgroups and allow for the generalizability of the results, we applied weights provided in the SDP^24^ in all bivariate and multivariate analyses.

### 2.2 Linear Blinder‐Oaxaca decomposition

We began with ordinary least squares (OLS) regression to estimate the log of hourly inflation‐adjusted net income as a function of dentist race and ethnicity and set observable dentist characteristics outlined in the previous section. We ran separate models pooling dentists of all races and ethnicities, models for each race and ethnicity, and then comparisons between White dentists to each of the other minoritized dentist populations.

We employed the commonly used Blinder^25^ and Oaxaca^26^ methodology to decompose the mean differences in the outcome variable between the two groups into “explained” and “unexplained” components, also referred to as a two‐fold decomposition. In the labor economics literature, the “explained” component, also referred to as the “composition” effect, captures the differential in earnings that is due to observable average differences between minoritized and White dentists. For example, if specialists typically earn more than GP dentists and a higher proportion of Whites are specialists, this could explain a portion of the observable gap in earnings between White and minoritized dentists. The “unexplained” component, also referred to as the “wage structure” effect or the effect due to discrimination, captures differences in the relationship between a given characteristic and earnings for White and minoritized dentists.^27^ For example, assuming a return to education should be the same across racial and ethnic groups, if White dentists have a greater return to experience than Asian dentists, this would be captured in the “unexplained” component of the Blinder‐Oaxaca decomposition. In our main results, we present a decomposition based on reference coefficients from a pooled regression model with a group indicator (e.g., White vs. minoritized dentists), as well as comparing each racial and ethnic group of dentists to White dentists as the referent group as done in previous research.^28^, ^29^, ^30^ We also test for counterfactuals, such that the reference coefficients are derived from an OLS regression model from only the White dentist group. In our results, the total “explained” and “unexplained” components of the decomposition are presented as a percentage of the total difference in earnings between White and minoritized dentists for ease of comparison across models.

We also ran separate Blinder‐Oaxaca decompositions by experience category. We compared the earnings of minoritized dentists with 20 years of experience or less to White dentists with 20 years of experience or less. We also estimated a Blinder‐Oaxaca decomposition comparing earnings of minoritized dentists with more than 20 years of experience to White dentists with more than 20 years of experience. We chose this cutoff since the return to experience begins to decrease for dentists after 20–25 years based on our observation of the data.

### 2.3 Oaxaca decomposition with re‐centered influence functions

Given that dentist earnings vary within subgroups, we used a methodology developed by Firpo, Fortin and Lemieux (2009)^31^ called a re‐centered influence function (RIF) to perform a Blinder‐Oaxaca style decomposition for other statistics besides the mean, such as a quantile or specific percentile. Conditional on observables, we estimated an unconditional quantile regression model based on the RIF, where the density function is estimated via kernel density methods, and an indicator function is equal to 1 if the outcome variable is at or below the quantile of interest. Using the RIF as the dependent variable, we conducted a Blinder‐Oaxaca decomposition to examine the White versus minoritized dentist earnings gap for the 10th, 50th, and 90th percentiles. We estimated the linear and RIF Blinder‐Oaxaca decompositions using the Oaxaca command in Stata 17.1.^32^ Estimating equations and technical details behind the linear and RIF Oaxaca decompositions are presented in the technical appendix.

## 3 RESULTS

### 3.1 Summary statistics

Our sample is 83.0% White, 2.0% Black, 3.1% Hispanic, 10.3% Asian, and 1.6% other races (Table A1). On average, White dentists earn more ($254,860) than Asian ($202,967), Black ($170,097), and Hispanic ($198,565) dentists. White dentists are older (50.9 years) and less likely to be female (14.5%) than dentists who are Asian (aged 45.1 years; female 35.2%), Black (aged 47.7 years; female 39.6%), and Hispanic (aged 47.3; female 34.0%). Also, compared to White (1.6%) and Black (1.1%) dentists, Asian (13.1%) and Hispanic (26.4%) dentists are more likely to be educated in a foreign dental school. The hours worked per year and per week are similar across all racial and ethnic categories.

On average, about one‐fifth of Black dentists' patients are on Medicaid compared to 6.4% of White dentists' patients. Asian and Hispanic dentists also have a larger percentage of Medicaid patients compared to White dentists. Only 17.7% of White dentists practice in ZIP codes that are less than a 50% White population compared to 59.5% of Asian, 58.3% of Black, and 53.7% of Hispanic dentists. A lower percentage of White dentists work in urban areas (87%) compared to minoritized dentists (over 96%). Summary statistics for experience subcategories (≤20 years and > 20 years) by race and ethnicity are shown in Table A2. Across all racial and ethnic categories, dentists with 20 years of experience or less are more likely to be female, more likely to participate in Medicaid or CHIP, and less likely to have graduated from a foreign dental school.

### 3.2 Trends in dentist earnings over time, distribution, and differentials of earnings by race and ethnicity

**FIGURE 1:**
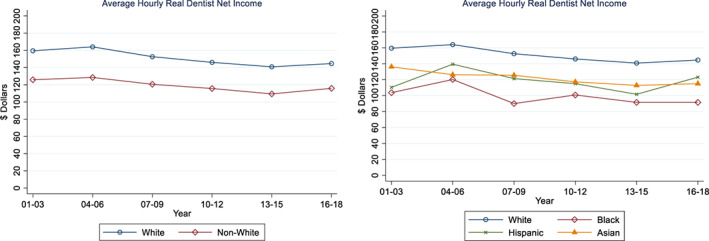
Trends in dentist earnings. SDP survey weights applied in all estimates. 2002–2019 American Dental Association Survey of Dental Practice. [Color figure can be viewed at wileyonlinelibrary.com]

The unadjusted ratio of earnings of White dentists to minoritized dentists remained fairly steady between 2001 and 2018. The gap in inflation‐adjusted hourly net income did not change appreciably between White and minoritized dentists (Figure 1). Between 2001 and 2018, hourly net income was about 22% lower for minoritized dentists compared to White dentists. Compared to White dentists, the unadjusted gap in hourly net income is 21% lower for Asian dentists, 32% lower for Black dentists, and 24% lower for Hispanic dentists (Table A3, Column 1). Controlling for observable factors, the gap in earnings between White and minoritized dentists narrows substantially but does not disappear. Compared to White dentists, the adjusted gap in hourly net income is 10.3% lower for Asian dentists, 24.2% lower for Black dentists, and 15.0% lower for Hispanic dentists (Table A3, Column 2). Results from OLS regressions whose coefficients are used as inputs in the Blinder‐Oaxaca decomposition are presented in Table A3. Results for OLS regressions for experience subcategories (≤20 years and >20 years) by race and ethnicity are shown in Table A4.

**FIGURE A1:**
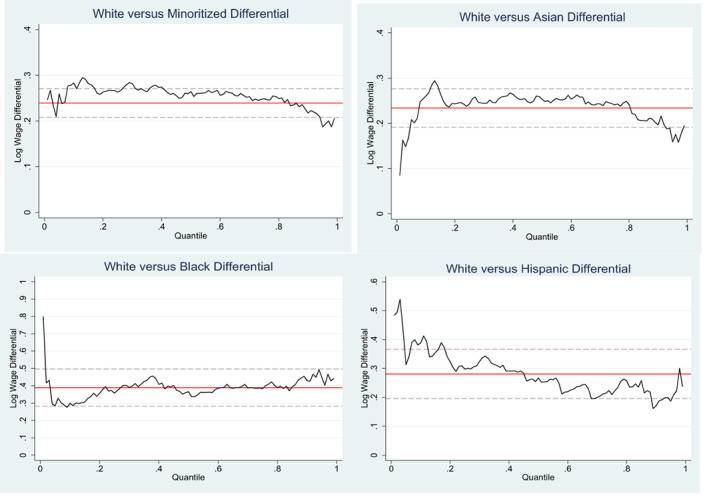
Race and ethnic differentials in log of inflation‐adjusted hourly net income. Red line represents raw differential in log hourly net income between White and minority dentists. Dashed lines represent 95% confidence intervals. 2002–2019 American Dental Association Survey of Dental Practice. [Color figure can be viewed at wileyonlinelibrary.com]

Examining the differential in the log of hourly dentist net income by quantile for White versus minoritized dentists (Figure A1), the earnings differential between White versus minoritized dentists hovers within the 95% confidence interval in the 15–85 interquantile range. Only at the highest quantiles (>95th percentile) does the gap in the log of hourly earnings between White and minoritized dentists narrow. The differential in the log of hourly earnings persists between White and Black dentists, and White and Hispanic dentists for nearly all quantiles except at the lowest quantiles where the gap widens.

### 3.3 Linear Blinder‐Oaxaca decomposition

Based on a pooled Blinder‐Oaxaca model of dentists from all racial and ethnic categories, observables account for 44.1% of the gap in log earnings between White and minoritized dentists, while the “unexplained” gap accounts for 55.9% of the gap (Table 1). Mean differences in race and ethnicity of a dentist's ZIP code, specialty, urbanicity, location of dental school (foreign or domestic), gender, and the percentage of patients with Medicaid/CHIP account for much of the “explained gap” between White and minoritized dentists. In a model comparing White to Asian dentists, the “explained” gap in log earnings is 0.135, which represents 57.7% of the total log earnings gap of which gender alone accounts for 34.1% of the “explained” gap. Receiving training from a foreign dental school accounts for 16.3% of the “explained” gap in earnings between White and Asian dentists. Other statistically significant factors contributing to the “explained” gap between Asian and White dentists include specialty, gender, urbanicity, the racial and ethnic makeup of the practice ZIP code, and the patient mix of a dentist's patients.

**TABLE 1:** Linear Blinder‐Oaxaca decomposition of White versus minoritized dentists

Variables	White versus Minoritized		White versus Asian		White versus Hispanic		White versus Black	
	Explained	Unexplained	Explained	Unexplained	Explained	Unexplained	Explained	Unexplained
Experience	−0.005	0.001	−0.002	−0.017	−0.012***	−0.125	−0.008	0.339*
	(0.003)	(0.063)	(0.004)	(0.066)	(0.004)	(0.178)	(0.006)	(0.202)
Gender	0.042***	−0.046***	0.046***	−0.059***	0.048***	−0.045	0.066***	−0.002
	(0.004)	(0.015)	(0.004)	(0.020)	(0.006)	(0.034)	(0.009)	(0.056)
Specialty	0.010***	−0.002	0.011**	0.001	0.005	0.0003	0.019*	0.008
	(0.004)	(0.007)	(0.005)	(0.009)	(0.008)	(0.016)	(0.010)	(0.020)
Race and ethnicity of ZIP code	0.020***	−0.001	0.023***	−0.010	0.022***	0.064	0.023***	−0.164**
	(0.006)	(0.023)	(0.007)	(0.033)	(0.006)	(0.055)	(0.007)	(0.083)
Urbanicity	0.003**	−0.005	0.003**	−0.006	0.002	0.0003	0.003*	−0.010
	(0.002)	(0.004)	(0.002)	(0.005)	(0.002)	(0.008)	(0.002)	(0.009)
School (foreign or domestic)	0.020***	−0.022***	0.022***	−0.029***	0.069***	−0.049*		
	(0.005)	(0.007)	(0.005)	(0.010)	(0.014)	(0.027)		
Year	−0.006	0.160	−0.005	0.418	−0.007	0.110	−0.006	−0.716
	(0.004)	(0.252)	(0.004)	(0.402)	(0.007)	(0.588)	(0.004)	(0.521)
BEA region	−0.004	0.135	−0.003	0.098	−0.002	0.017	0.009**	0.827
	(0.005)	(0.115)	(0.007)	(0.146)	(0.004)	(0.294)	(0.004)	(0.680)
Percentage with Medicaid/CHIP	0.015***	−0.005	0.021***	0.015	0.010***	−0.023	0.022***	−0.072
	(0.003)	(0.013)	(0.004)	(0.018)	(0.003)	(0.028)	(0.006)	(0.049)
Regional CPI	0.015**	−0.579	0.019**	−1.716	0.019**	−0.937	−0.008	2.071
	(0.007)	(1.143)	(0.009)	(1.764)	(0.008)	(2.816)	(0.007)	(2.173)
Constant		0.508		1.403		1.114		−2.011
		(1.000)		(1.514)		(2.484)		(2.123)
Unadjusted log earnings gap	0.254***		0.234***		0.281***		0.389***	
	(0.022)	%	(0.027)	%	(0.050)	%	(0.067)	%
Total explained by model	0.112***	44.1%	0.135***	57.7%	0.154***	54.8%	0.120***	30.8%
	(0.011)		(0.013)		(0.020)		(0.019)	
Unexplained log earnings gap	0.143***	55.9%	0.098***	42.3%	0.127**	45.2%	0.270***	69.2%
	(0.023)		(0.028)		(0.050)		(0.067)	
Number of observations	22,086		20,898		19,737		19,481	
White observations	19,184		19,184		19,184		19,184	
Minoritized observations	2902		1714		553		297	

*Note*: SDP survey weights applied in all estimates. The indicator for foreign trained dentist is excluded from the model for Black dentists due to the small sample cell size. Robust standard errors are in parentheses. Reference coefficients based on the pooled regression model. ***p < 0.01, **p < 0.05, *p < 0.1. 2002–2019 American Dental Association Survey of Dental Practice.

Abbreviations: BEA, Bureau of economic analysis; CHIP, children's health insurance program; CPI, consumer price index.

When comparing White to Hispanic dentists, experience has a negative and statistically significant “explained” effect on the log earnings gap. In our sample, White dentists have a higher average level of experience compared to Hispanic dentists. Also, since most of our sample is older and more likely to be White and given that the return to experience for a dentist declines after 20 years, it is expected that experience has a negative “explained” effect on the earnings gap between White and Hispanic dentists. The “explained” gap in log earnings between White and Hispanic dentists is 0.154, which accounts for 54.8% of the total log earnings gap.

The “explained” gap between White and Black dentists accounts for 30.8% of the total log earnings gap. Gender, the racial and ethnic mix of a dentist practice's ZIP code, BEA region and patient mix are statistically significant factors that account for the “explained” log earnings gap between White and Black dentists. The patient mix between publicly and privately insured patients accounts for 5.7% of the total gap and 18.3% of the “explained” log earnings gap between Black and White dentists. Most of the total earnings gap between White and Black dentists is “unexplained” by observables.

To test for counterfactuals, we conducted Blinder‐Oaxaca linear regression models where we assumed all of the characteristics of White dentists were taken on by minoritized dentists (Table A5). When minoritized dentists were assumed to have the same characteristics as White dentists (in other words, the White reference coefficients are used in the Blinder‐Oaxaca decomposition), the total log earnings gap “explained” by observables is larger (Table A5) than the pooled model specification. The coefficients in the “explained” component of the Blinder‐Oaxaca decomposition are close in magnitude and identical in sign when White reference coefficients are used. Our results are also qualitatively similar when we estimate an unweighted Blinder‐Oaxaca Decomposition (Table A6), although the percentage of the earnings gap “explained” by observables is lower than in the main specification (Table 1).

To account for the possibility that some racial groups in some ZIP codes changed over time, we also do a robustness check by estimating a Blinder‐Oaxaca decomposition comparing White to minoritized dentists for data years 2015 through 2018, which corresponds to years of data we used from the ACS to identify whether a ZIP code is less than 50% White (Table A7). Under this specification, the percent “explained” by observables goes up slightly to 51.3%, and the results are qualitatively and quantitatively similar to the main specification (Table 1), particularly on the explained coefficients on gender, race, and ethnicity of ZIP code, patient mix, and regional CPI.

### 3.4 Linear Blinder‐Oaxaca decomposition by years of experience

Results from the linear Blinder‐Oaxaca earning decomposition model stratified by years of experience (≤20 years and >20 years) are shown in Table A8. Among dentists with ≤20 years' experience, the percentage of the log earnings gap explained by observables is 51.8% in the White versus Asian comparison, 44.3% in the White versus Hispanic comparison, and 34.3% in the White versus Black comparison. The percentage of the log earnings gap explained by observables is 70.1% in the White versus Asian comparison, 83.3% in the White versus Hispanic comparison, and 26.8% in the White versus Black comparison for dentists with >20 years' experience. For dentists with >20 years' experience, the “explained” coefficient on experience is negative and statistically significant in the White versus Asian dentist and White versus Hispanic dentist comparisons. This is expected, given that White dentists have more experience on average than Asians and Hispanics and the return to experience on dentist earnings turns negative after 20 years of tenure.

Across the experience subcategories, the coefficient on gender and the racial and ethnic mix of the dentist's practice ZIP code in the “explained” component of the decomposition is positive and statistically significant in most racial and ethnic comparisons. For dentists with ≤20 years' experience, gender accounts for 28.1% of the “explained” gap between White and Asian dentists, 23.0% of the “explained” gap between White and Hispanic dentists, and 58.3% of the “explained” gap between White and Black dentists. Gender accounts for 25.3% of the “explained” gap between White and Asian dentists, 28.6% of the “explained” gap between Whites and Hispanic dentists, and 39.7% of the “explained” gap between White and Black dentist with >20 years of experience .

For dentists with ≤20 years of experience, dental school location (foreign or domestic) accounts for 5.3% of the “explained” gap between White and Asian dentists and 33.8% of the “explained” gap between White and Hispanic dentists. Dental school location accounts for a larger percentage of the “explained” gap between White and Asian dentists (33.3%) and White and Hispanic dentists (52.9%) with >20 years of experience.

For dentists with >20 years of experience, private/Medicaid insurance patient mix accounts for 18.5% of the “explained” gap between White and Asian dentists, 10.0% of the “explained” gap between White and Hispanic dentists, and 33.3% of the “explained” gap between White and Black dentists. Patient insurance status was not a statistically significant factor in explaining the log wage differential for any of the comparisons among dentists with 20 years of experience or less.

### 3.5 Unconditional quantile Blinder‐Oaxaca decomposition

Using RIFs, for White versus Asian dentists (Table 2), Whites versus Hispanic dentists (Table 3), and White versus Black dentists (Table 4), we estimated an unconditional quantile Blinder‐Oaxaca decomposition at the 10th, 50th, and 90th percentiles of the log earnings distribution. The total unadjusted gap in log earnings between White and Asian dentists decreases from 0.260 at the 10th percentile to 0.195 at the 90th percentile (Table 2). The percentage of the gap in earnings “explained” by observables in the White versus Asian comparison decreases from 92.7% at the 10th percentile to 46.7% at the 90th percentile. At the 10th percentile, gender accounts for 29.9% of the “explained” gap compared to 37.4% at the 90th percentile. Dental school accounts for 34.4% of the “explained” gap in earnings between White and Asian dentists at the 10th percentile and 26.4% at the 90th percentile. Years of experience has a negative and statistically significant association on the “explained” gap between White and Asian dentists at the 10th percentile but then becomes statistically insignificant at the 50th and 90th percentiles. This suggests that the “explained” earnings gap attributable to dentist tenure mainly occurs at the lower end of the dentist earnings distribution.

**TABLE 2:** White versus Asian RIF Blinder‐Oaxaca decomposition

Variables	RIF 10th percentile		RIF 50th percentile		RIF 90th percentile	
	Explained	Unexplained	Explained	Unexplained	Explained	Unexplained
Experience	−0.018**	−0.074	0.002	−0.012	0.003	−0.063
	(0.008)	(0.132)	(0.006)	(0.070)	(0.006)	(0.069)
Gender	0.072***	−0.140***	0.058***	−0.053**	0.034***	−0.015
	(0.011)	(0.037)	(0.007)	(0.022)	(0.005)	(0.027)
Specialty	0.007**	0.012	0.010**	0.000	0.017**	0.002
	(0.003)	(0.010)	(0.004)	(0.008)	(0.007)	(0.014)
Race and ethnicity of ZIP code	0.037**	−0.000	0.023***	−0.014	0.010	−0.022
	(0.016)	(0.058)	(0.009)	(0.036)	(0.009)	(0.043)
Urbanicity	0.007*	−0.003	0.004*	−0.004	−0.004	−0.011
	(0.003)	(0.005)	(0.002)	(0.004)	(0.002)	(0.008)
School (foreign or domestic)	0.083***	−0.070**	0.028***	−0.042***	0.024***	−0.018
	(0.022)	(0.030)	(0.007)	(0.013)	(0.005)	(0.012)
Year	−0.016*	1.024	−0.009	0.559	−0.012**	0.753**
	(0.009)	(0.664)	(0.005)	(0.342)	(0.006)	(0.361)
BEA region	0.009	−0.079	−0.012	0.017	−0.019*	0.072
	(0.017)	(0.189)	(0.010)	(0.171)	(0.010)	(0.254)
Percentage with medicaid/CHIP	0.026***	0.006	0.025***	0.011	0.003	0.008
	(0.010)	(0.034)	(0.006)	(0.020)	(0.005)	(0.023)
Regional CPI	0.034*	−5.167	0.029**	−2.127	0.035***	−2.679**
	(0.021)	(3.336)	(0.012)	(1.646)	(0.012)	(1.358)
Constant		4.511		1.760		2.077*
		(2.881)		(1.425)		(1.161)
Unadjusted log earnings gap	0.260***		0.253***		0.195***	
	(0.041)	%	(0.028)	%	(0.033)	%
Total explained by model	0.241***	92.7%	0.159***	62.8%	0.091***	46.7%
	(0.034)		(0.016)		(0.015)	
Unexplained log earnings gap	0.020	7.3%	0.094***	37.2%	0.104***	53.3%
	(0.055)		(0.031)		(0.035)	
Number of observations	20,898		20,898		20,898	
White observations	19,184		19,184		19,184	
Asian observations	1714		1714		1714	

*Note*: SDP survey weights applied in all estimates. Robust standard errors are in parentheses. Reference coefficients based on all‐White sample. ***p < 0.01, **p < 0.05, *p < 0.1. 2002–2019 American Dental Association Survey of Dental Practice.

Abbreviations: BEA, Bureau of economic analysis; CHIP, children's health insurance program; CPI, consumer price index; RIF, re‐centered influence function.

**TABLE 3:** White versus Hispanic RIF Blinder‐Oaxaca decomposition

Variables	RIF 10th percentile		RIF 50th percentile		RIF 90th percentile	
	Explained	Unexplained	Explained	Unexplained	Explained	Unexplained
Experience	−0.021***	−0.208	−0.012*	−0.189	−0.006	−0.018
	(0.006)	(0.318)	(0.007)	(0.178)	(0.005)	(0.217)
Gender	0.067***	−0.103	0.054***	−0.058	0.032***	−0.028
	(0.014)	(0.064)	(0.009)	(0.039)	(0.006)	(0.041)
Specialty	0.003	−0.032	0.005	−0.011	0.008	0.052*
	(0.005)	(0.021)	(0.007)	(0.018)	(0.012)	(0.027)
Race and ethnicity of ZIP code	0.032**	0.171*	0.020***	0.019	0.009	0.031
	(0.014)	(0.102)	(0.008)	(0.064)	(0.008)	(0.064)
Urbanicity	0.006*	0.006	0.004*	−0.001	−0.003	−0.000
	(0.003)	(0.011)	(0.002)	(0.008)	(0.002)	(0.015)
School (foreign or domestic)	0.180***	−0.132*	0.061***	−0.021	0.052***	−0.020
	(0.049)	(0.068)	(0.016)	(0.039)	(0.011)	(0.043)
Year	−0.020	0.131	−0.010	0.846	−0.014	−0.783
	(0.016)	(0.842)	(0.009)	(0.655)	(0.010)	(0.976)
BEA region	0.011	0.107	−0.006	0.115	−0.006	−0.383
	(0.008)	(0.404)	(0.005)	(0.317)	(0.005)	(0.449)
Percentage with Medicaid/CHIP	0.016**	−0.081**	0.016***	−0.015	0.002	−0.039
	(0.007)	(0.041)	(0.005)	(0.033)	(0.003)	(0.041)
Regional CPI	0.028	−0.694	0.024**	−2.989	0.029**	2.719
	(0.018)	(3.534)	(0.011)	(2.879)	(0.012)	(5.081)
Constant		0.914		2.403		−1.467
		(3.100)		(2.522)		(4.525)
Unadjusted log earnings gap	0.380***		0.255***		0.165**	
	(0.086)	%	(0.056)	%	(0.065)	%
Total explained by model	0.303***	79.7%	0.156***	61.2%	0.102***	61.8%
	(0.056)		(0.022)		(0.020)	
Unexplained log earnings gap	0.077	20.3%	0.100*	38.8%	0.063	38.2%
	(0.102)		(0.057)		(0.065)	
Number of observations	19,737		19,737		19,737	
White observations	19,184		19,184		19,184	
Hispanic observations	553		553		553	

*Note*: SDP survey weights applied in all estimates. Robust standard errors are in parentheses. Reference coefficients based on all‐White sample. ***p < 0.01, **p < 0.05, *p < 0.1. 2002–2019 American Dental Association Survey of Dental Practice.

Abbreviations: BEA, Bureau of economic analysis; CHIP, children's health insurance program; CPI, consumer price index; RIF, re‐centered influence function.

**TABLE 4:** White versus Black RIF Blinder‐Oaxaca decomposition

Variables	RIF 10th percentile		RIF 50th percentile		RIF 90th percentile	
	Explained	Unexplained	Explained	Unexplained	Explained	Unexplained
Experience	−0.016**	0.596**	−0.005	0.435**	0.003	−0.301
	(0.008)	(0.251)	(0.009)	(0.196)	(0.007)	(0.192)
Gender	0.090***	0.080	0.071***	0.013	0.042***	−0.102*
	(0.019)	(0.100)	(0.013)	(0.054)	(0.008)	(0.059)
Specialty	0.012**	−0.006	0.018**	0.020	0.030**	0.043
	(0.006)	(0.020)	(0.009)	(0.019)	(0.015)	(0.026)
Race and ethnicity of ZIP code	0.044***	−0.214*	0.025***	−0.242***	0.012	−0.072
	(0.017)	(0.112)	(0.009)	(0.080)	(0.009)	(0.067)
Urbanicity	0.008**	−0.007	0.005**	−0.015	−0.003	−0.008
	(0.004)	(0.007)	(0.002)	(0.010)	(0.002)	(0.011)
Year	−0.010	−0.200	−0.007	−1.474***	−0.006	−0.407
	(0.009)	(0.606)	(0.005)	(0.562)	(0.007)	(0.534)
BEA region	0.008	0.358	0.010*	1.155***	0.006	0.946
	(0.009)	(0.440)	(0.006)	(0.319)	(0.006)	(0.951)
Percentage with Medicaid/CHIP	0.042***	−0.094	0.036***	−0.122***	0.006	−0.069
	(0.015)	(0.061)	(0.009)	(0.047)	(0.007)	(0.065)
Regional CPI	−0.010	1.161	−0.008	3.696	−0.010	1.196
	(0.011)	(2.959)	(0.009)	(2.384)	(0.011)	(2.503)
Constant		−1.551		−3.251		−0.879
		(2.743)		(2.209)		(2.347)
Unadjusted log earnings gap	0.291***		0.360***		0.427***	
	(0.095)	%	(0.068)	%	(0.074)	%
Total explained by model	0.167***	57.4%	0.145***	40.3%	0.080***	18.7%
	(0.034)		(0.025)		(0.023)	
Unexplained log earnings gap	0.124	42.6%	0.215***	59.7%	0.347***	81.3%
	(0.100)		(0.072)		(0.075)	
Number of observations	19,481		19,481		19,481	
White observations	19,184		19,184		19,184	
Black observations	297		297		297	

*Note*: SDP survey weights applied in all estimates. Indicator for foreign trained dentist is excluded from the model for Black dentists due to the small sample cell size. Robust standard errors are in parentheses. Reference coefficients based on all‐White sample. ***p < 0.01, **p < 0.05, *p < 0.1. 2002–2019 American Dental Association Survey of Dental Practice.

Abbreviations: BEA, Bureau of economic analysis; CHIP, children's health insurance program; CPI, consumer price index; RIF, re‐centered influence function.

The total unadjusted gap in log earnings between White and Hispanic dentists decreases from 0.380 at the 10th percentile to 0.165 at the 90th percentile (Table 3). The percentage of the gap in earnings “explained” by observables in the White versus Hispanic dentist comparison decreases from 79.7% at the 10th percentile to 61.8% at the 90th percentile. The patient mix between publicly and privately insured patients has a positive statistically significant effect on the “explained” component of the decomposition at the 10th and 50th percentiles. This suggests that patient mix has a bigger association with the total gap in earnings between White and Hispanic dentists in the lower half of the earnings distribution. The association of dental school on the total earnings gap between White and Hispanic dentists decreases as one moves from the 10th percentile to the 90th percentile.

The total unadjusted gap in log earnings between White and Black dentists increases from 0.291 at the 10th percentile to 0.427 at the 90th percentile (Table 4). The percentage of the gap in earnings “explained” by observables in the comparison of White versus Black dentists decreases from 57.4% at the 10th percentile to 18.7% at the 90th percentile. As in the comparisons between White and Asian dentists and White and Hispanic dentists, this suggests that a large component of the earnings gap between White and Black dentists is not captured by observables at the highest earnings quantiles. As in the comparisons of White versus Asian and White versus Hispanic dentists, experience only has a statistically significant association on the “explained” component of the decomposition at the 10th percentile for the comparison of White and Black dentists. The racial and ethnic composition of dentist practice ZIP code and percentage of publicly insured patients have a positive and statistically significant association on the “explained” component of the decomposition in the gap in log earnings between White and Black dentists at the 10th and 50th percentiles before becoming statistically insignificant at the 90th percentile. The percentage of the “explained” gap captured by patient mix, as measured by the percentage of patients that are Medicaid/CHIP insured, is about 25% at the 10th and 50th percentile, which suggests that patient mix has a significant association with the earnings gap between White and Black dentists at the lower half of the earnings distribution.

Our results are also qualitatively similar when we estimate an unweighted unconditional quantile Blinder‐Oaxaca decomposition comparing White to Asian dentists (Table A9), White to Hispanic dentists (Table A10), and White to Black dentists (Table A11). However, in all racial and ethnic comparisons at the 10th, 50th, and 90th percentiles, the percent “explained” by observables is lower than in the main specification (Tables 2, 3, 4).

## 4 DISCUSSION

Unlike the convergence in earnings between male and female dentists,^33^ the gap in hourly dentist net income between White dentists and minoritized dentists has not narrowed between 2001 and 2018. Compared to other minoritized dentists, Black dentists have the largest earnings disparities relative to White dentists, with the majority of the earnings gap “unexplained” or not captured by observable characteristics. The excess percentage of the “unexplained” component for Black dentists relative to other minoritized dentists may be indicative of discrimination that Black dentists may experience directly and the systemic factors contributing to this discrimination.

Previous studies examining labor market discrimination between racial and ethnic groups in the general worker population concluded that differences in skills and education accounted for the gap in earnings between groups, not discrimination.^17^, ^34^ In our analysis, however, we examined the gap in dentist earnings among individuals who all graduated from dental school and presumably have a similar level of skills. One may argue that specialists have a higher level of skill and education than GP dentists, but we included an indicator variable for a specialist dentist in our specifications and still found that most of the gap in dentist earnings between groups was “unexplained” when comparing White and Black dentists. Our study found that training at a foreign dental school significantly contributed to the “explained” wage gap between White and Asian, as well as White and Hispanic dentists (note that the sample of foreign‐trained Black dentists was too small to analyze in this study), with a larger detrimental effect on the wages of Hispanic dentists. While we had information on the school from which dentists graduated, we do not have a universally accepted measure of the quality^35^ of domestic and foreign schools, such as a dental school ranking, which could potentially influence wages through differences in productivity or job opportunities.^36^ We also did not control for the language spoken or fluency of dentists, which literature suggests has contributed to discrimination^37^, ^38^ by patients and supervisors against foreign‐trained health care workers with regards to, for example, patient load and promotions, which again influence wages.

A concern motivating this study is the lagging diversity of the dentist workforce relative to the increasingly diverse patient population, in part due to the growing use of dental services by Medicaid patients.^10^ Our study found that caring for a higher percentage of Medicaid patients significantly contributes to the “explained” wage gap between White and minoritized dentists as minoritized dentists, but only among those who had 20 or more years of experience. A recent study found that younger dentists and minoritized dentists are more likely to care for Medicaid patients than older and White dentists,^39^ which may contribute to lower wages through lower reimbursement rates relative to private health insurance. In considering whether the higher Medicaid patient panel is related to dentists choosing to care for these patients or that Medicaid patients are the only ones that these dentists could attract to their practice, our findings show that dentists practicing in ZIP codes with greater racial and ethnic diversity further added to the wage differential between White and minoritized dentists regardless of years of experience. This finding suggests that neighborhood effects may be separate from the effects of the insurance mix.

There are a number of limitations in our study. The measure of net income in the SDP not only includes salary but also includes bonuses, dividends, and commissions. Unfortunately, the survey data do not allow for bonuses, dividends and commissions to be separated from wage income, nor the use of these other components of net income as additional independent variables. Although the level of net income from the SDP is upwardly biased relative to other measures of salary income as measured by the Bureau of Labor Statistics,^40^ we have no information to assume that this bias would affect the differential in net income between White and minoritized dentists. Second, we do not have information available on differences in characteristics of SDP respondents versus nonrespondents, although we used established weights to support the generalizability of the results and found little difference in results in our unweighted analyses. Third, the SDP does not include all factors that may further account for the unexplained portion of the wage gap, such as marital status, whether a dentist respondent has a parent who was a dentist nor the full educational history of the dentist before dental school. Related, we do not have information on whether the location of a dentist's primary practice was subject to historical redlining or other discriminatory practices, which could affect the current level of the earnings disparity between White and minoritized dentists.

## 5 CONCLUSION

This study provides the first comprehensive and longitudinal examination of wage disparities among dentists by race and ethnicity. The persistent wage disparities experienced by minoritized dentists are concerning when we are seeing an increasingly minoritized patient population that may have benefited from receiving care from a racially and ethnically concordant provider population. With the growing use of dental services among Medicaid patients, policy makers should ensure parity between Medicaid and private health insurance reimbursement rates to ensure that dentists and mostly minoritized dentists who take on higher Medicaid loads are not systematically experiencing lower wages. If this wage difference persists, dentists may be discouraged in caring for an already underserved population. Additionally, support and protections may be needed for foreign‐trained dentists to ensure that they are not discriminated against by employers, and thus, experience lower wages. While this study provides important insight as to the “explained” contributors to wage disparities between White and minoritized dentists, further work is needed to understand the system‐wide factors that may be contributing to large “unexplained” wage gaps experienced by minoritized dentists, particularly Black dentists to ensure a diverse dentist workforce into the future.

## FUNDING INFORMATION

No funding to report.

## Supporting information


**Appendix S1.** Supporting Information.

## APPENDIX A

**TABLE A1:** Summary statistics by race and ethnicity

Variables	All	White	Minoritized	Asian	Black	Hispanic
Observations	22,086	19,184	2902	1714	297	553
Real dentist net income (2018$)	245,600	254,860	200,346	202,967	170,097	198,565
	(173,898)	(177,622)	(146,228)	(149,668)	(120,690)	(142,867)
Real dentist hourly net income (2018$)	145.8	151.4	118.7	121.1	101.1	117.6
	(103.5)	(105.9)	(85.97)	(89.33)	(66.43)	(84.08)
Age	50.09	50.92	46.04	45.12	47.71	47.28
	(11.47)	(11.44)	(10.73)	(10.59)	(11.98)	(10.30)
Experience 1–5 years	0.0806	0.0734	0.116	0.136	0.0810	0.0947
	(0.272)	(0.261)	(0.320)	(0.343)	(0.273)	(0.293)
Experience 6–10 years	0.110	0.0977	0.168	0.174	0.189	0.154
	(0.312)	(0.297)	(0.374)	(0.379)	(0.393)	(0.361)
Experience 11–15 years	0.115	0.104	0.169	0.178	0.175	0.141
	(0.319)	(0.305)	(0.375)	(0.383)	(0.381)	(0.348)
Experience 16–20 years	0.122	0.113	0.163	0.161	0.122	0.163
	(0.327)	(0.317)	(0.370)	(0.367)	(0.328)	(0.369)
Experience 21–25 years	0.144	0.145	0.140	0.137	0.128	0.152
	(0.351)	(0.352)	(0.347)	(0.344)	(0.335)	(0.359)
Experience 26–30 years	0.154	0.164	0.105	0.0997	0.0936	0.138
	(0.361)	(0.370)	(0.306)	(0.300)	(0.292)	(0.345)
Experience 31–35 years	0.126	0.138	0.0702	0.0522	0.107	0.0971
	(0.332)	(0.345)	(0.255)	(0.222)	(0.310)	(0.296)
Experience 36–40 years	0.0848	0.0935	0.0421	0.0338	0.0673	0.0401
	(0.279)	(0.291)	(0.201)	(0.181)	(0.251)	(0.196)
Experience 41+ years	0.0649	0.0725	0.0273	0.0275	0.0360	0.0214
	(0.246)	(0.259)	(0.163)	(0.164)	(0.187)	(0.145)
Female	0.178	0.145	0.340	0.352	0.396	0.340
	(0.382)	(0.352)	(0.474)	(0.478)	(0.490)	(0.474)
ZIP code <50% White population	0.241	0.177	0.554	0.595	0.583	0.537
	(0.428)	(0.382)	(0.497)	(0.491)	(0.494)	(0.499)
Urban	0.886	0.869	0.967	0.972	0.975	0.965
	(0.318)	(0.337)	(0.179)	(0.164)	(0.156)	(0.185)
Dentist trained in foreign dental school	0.0370	0.0156	0.141	0.131	0.0111	0.264
	(0.189)	(0.124)	(0.348)	(0.337)	(0.105)	(0.441)
Hours worked per week	36.08	35.97	36.64	36.41	36.55	36.98
	(7.502)	(7.332)	(8.259)	(8.138)	(8.470)	(8.472)
Hours worked per year	1728	1726	1735	1720	1716	1760
	(398.0)	(388.6)	(441.4)	(442.0)	(443.7)	(434.7)
General practice dentist	0.817	0.814	0.833	0.836	0.853	0.824
	(0.387)	(0.389)	(0.373)	(0.371)	(0.355)	(0.381)
Dentist participates in Medicaid or CHIP	0.386	0.367	0.481	0.488	0.562	0.429
	(0.487)	(0.482)	(0.500)	(0.500)	(0.497)	(0.495)
Percentage of patients in Medicaid or CHIP	7.910	6.393	15.32	15.89	19.20	12.45
	(17.25)	(15.03)	(24.12)	(24.53)	(26.18)	(21.88)
New england BEA region	0.0460	0.0502	0.0258	0.0306	0.0111	0.0117
	(0.210)	(0.218)	(0.159)	(0.172)	(0.105)	(0.108)
Middle east BEA region	0.166	0.167	0.162	0.155	0.332	0.109
	(0.372)	(0.373)	(0.368)	(0.362)	(0.472)	(0.312)
Great lakes BEA region	0.161	0.177	0.0833	0.0626	0.113	0.0721
	(0.368)	(0.382)	(0.276)	(0.242)	(0.317)	(0.259)
Plains BEA region	0.0706	0.0814	0.0180	0.0106	0.0197	0.0284
	(0.256)	(0.273)	(0.133)	(0.102)	(0.139)	(0.166)
Southeast BEA region	0.204	0.213	0.161	0.100	0.306	0.260
	(0.403)	(0.410)	(0.367)	(0.300)	(0.462)	(0.439)
Southwest BEA region	0.100	0.0988	0.107	0.0893	0.150	0.142
	(0.300)	(0.298)	(0.309)	(0.285)	(0.357)	(0.350)
Far West/Rocky mountain BEA region	0.251	0.212	0.443	0.552	0.0691	0.376
	(0.434)	(0.409)	(0.497)	(0.497)	(0.254)	(0.485)
Regional CPI	215.5	214.6	219.7	221.0	212.8	219.8
	(23.70)	(23.73)	(23.09)	(22.84)	(23.38)	(23.28)
White	0.830					
	(0.376)					
Black	0.0201					
	(0.140)					
Hispanic	0.0312					
	(0.174)					
Asian	0.103					
	(0.304)					
Other race	0.0157					
	(0.124)					

*Note*: SDP survey weights are applied in all estimates. Standard deviation in parentheses. 2002–2019 American Dental Association Survey of Dental Practice. 2016–2019 American Community Survey. United States Department of Agriculture. Bureau of Labor Statistics.

Abbreviations: BEA, Bureau of economic analysis; CHIP, children's health insurance program; CPI, consumer price index.

**TABLE A2:** Summary statistics by experience subcategory

Variables	White, 1–20	White, 21+	Minoritized, 1–20	Minoritized, 21+	Asian, 1–20	Asian, 21+	Black, 1–20	Black, 21+	Hispanic, 1–20	Hispanic, 21+
Real dentist net income	256,471	253,838	200,187	200,601	202,105	204,564	180,260	156,762	193,618	204,653
(2018$)	(181,917)	(174,847)	(147,870)	(143,617)	(150,648)	(147,943)	(122,041)	(118,052)	(149,136)	(134,819)
Real dentist hourly net income	147.7	153.7	117.4	120.8	119.7	123.7	105.8	95.03	113.6	122.6
(2018$)	(104.5)	(106.7)	(86.82)	(84.58)	(90.21)	(87.69)	(67.63)	(64.58)	(87.61)	(79.43)
Age	39.38	58.23	39.46	56.60	39.16	56.16	39.02	59.13	39.99	56.26
	(6.248)	(7.151)	(6.338)	(7.380)	(6.436)	(7.466)	(5.652)	(7.745)	(6.216)	(6.560)
Experience 1–5 years	0.189		0.188		0.209		0.143		0.172	
	(0.392)		(0.391)		(0.407)		(0.351)		(0.378)	
Experience 6–10 years	0.252		0.272		0.268		0.334		0.279	
	(0.434)		(0.445)		(0.443)		(0.473)		(0.449)	
Experience 11–15 years	0.267		0.275		0.275		0.309		0.255	
	(0.442)		(0.447)		(0.447)		(0.464)		(0.437)	
Experience 16–20 years	0.292		0.265		0.248		0.215		0.295	
	(0.455)		(0.442)		(0.432)		(0.412)		(0.457)	
Experience 21–25 years		0.236		0.364		0.392		0.297		0.338
		(0.425)		(0.481)		(0.489)		(0.458)		(0.474)
Experience 26–30 years		0.267		0.272		0.284		0.216		0.308
		(0.443)		(0.445)		(0.451)		(0.413)		(0.462)
Experience 31–35 years		0.225		0.183		0.149		0.248		0.217
		(0.418)		(0.387)		(0.356)		(0.433)		(0.413)
Experience 36–40 years		0.153		0.110		0.0965		0.156		0.0894
		(0.360)		(0.312)		(0.295)		(0.364)		(0.286)
Experience 41+ years		0.118		0.0712		0.0785		0.0833		0.0478
		(0.323)		(0.257)		(0.269)		(0.277)		(0.214)
Female	0.259	0.0721	0.411	0.226	0.421	0.226	0.517	0.237	0.399	0.267
	(0.438)	(0.259)	(0.492)	(0.418)	(0.494)	(0.419)	(0.501)	(0.427)	(0.491)	(0.443)
ZIP code <50% White	0.150	0.194	0.506	0.632	0.541	0.696	0.495	0.700	0.536	0.539
	(0.357)	(0.396)	(0.500)	(0.482)	(0.499)	(0.460)	(0.502)	(0.460)	(0.500)	(0.499)
Urban	0.870	0.869	0.970	0.962	0.975	0.966	0.981	0.966	0.964	0.966
	(0.337)	(0.338)	(0.169)	(0.192)	(0.155)	(0.180)	(0.135)	(0.181)	(0.188)	(0.182)
Dentist trained in foreign dental school	0.0137	0.0169	0.0724	0.252	0.0543	0.273		0.0257	0.196	0.348
	(0.116)	(0.129)	(0.259)	(0.434)	(0.227)	(0.446)		(0.159)	(0.398)	(0.477)
Hours worked per week	36.84	35.42	37.02	36.02	36.72	35.83	37.13	35.80	37.30	36.59
	(7.308)	(7.295)	(8.254)	(8.233)	(8.329)	(7.747)	(8.078)	(8.934)	(8.065)	(8.948)
Hours worked per year	1778	1693	1756	1700	1735	1691	1731	1696	1799	1713
	(384.3)	(387.6)	(452.7)	(420.6)	(460.4)	(404.7)	(427.3)	(465.3)	(440.6)	(423.6)
General practice dentist	0.807	0.818	0.836	0.830	0.840	0.828	0.825	0.889	0.828	0.820
	(0.395)	(0.386)	(0.371)	(0.376)	(0.367)	(0.377)	(0.381)	(0.315)	(0.379)	(0.385)
Dentist participates in Medicaid/CHIP	0.400	0.345	0.498	0.454	0.512	0.444	0.565	0.560	0.439	0.417
	(0.490)	(0.476)	(0.500)	(0.498)	(0.500)	(0.497)	(0.498)	(0.498)	(0.497)	(0.494)
Percentage of patients in Medicaid/CHIP	8.175	5.264	16.26	13.82	17.07	13.71	18.58	20.01	13.04	11.72
	(17.12)	(13.43)	(24.45)	(23.51)	(24.59)	(24.29)	(26.22)	(26.21)	(22.83)	(20.66)
New England BEA region	0.0423	0.0551	0.0296	0.0197	0.0332	0.0258	0.0115	0.0107	0.0175	0.00464
	(0.201)	(0.228)	(0.169)	(0.139)	(0.179)	(0.159)	(0.107)	(0.103)	(0.131)	(0.0681)
Middle East BEA region	0.137	0.186	0.170	0.149	0.175	0.118	0.288	0.389	0.113	0.104
	(0.344)	(0.389)	(0.376)	(0.356)	(0.380)	(0.322)	(0.454)	(0.489)	(0.318)	(0.306)
Great Lakes BEA region	0.148	0.196	0.0885	0.0749	0.0696	0.0496	0.114	0.111	0.0656	0.0800
	(0.355)	(0.397)	(0.284)	(0.263)	(0.255)	(0.217)	(0.319)	(0.315)	(0.248)	(0.272)
Plains BEA region	0.0937	0.0735	0.0149	0.0228	0.00869	0.0142	0.0190	0.0207	0.0208	0.0377
	(0.291)	(0.261)	(0.121)	(0.149)	(0.0928)	(0.118)	(0.137)	(0.143)	(0.143)	(0.191)
Southeast BEA region	0.237	0.198	0.186	0.119	0.141	0.0241	0.327	0.279	0.269	0.250
	(0.425)	(0.399)	(0.389)	(0.324)	(0.348)	(0.154)	(0.471)	(0.450)	(0.444)	(0.433)
Southwest EEA region	0.107	0.0938	0.120	0.0871	0.111	0.0492	0.183	0.105	0.125	0.163
	(0.309)	(0.292)	(0.325)	(0.282)	(0.314)	(0.216)	(0.388)	(0.308)	(0.331)	(0.370)
Far West/Rocky MTN BEA region	0.235	0.198	0.391	0.527	0.462	0.720	0.0569	0.0851	0.388	0.361
	(0.424)	(0.398)	(0.488)	(0.499)	(0.499)	(0.449)	(0.232)	(0.280)	(0.488)	(0.481)
Regional CPI	213.5	215.3	217.2	223.7	218.4	225.7	212.5	213.3	216.4	224.1
	(24.00)	(23.53)	(23.02)	(22.65)	(22.50)	(22.73)	(24.35)	(22.12)	(23.84)	(21.89)
Observations	5892	13,292	1509	1393	948	766	144	153	260	293

*Note*: SDP survey weights are applied in all estimates. Standard Deviation in parentheses. 2002–2019 American Dental Association Survey of Dental Practice. 2015–2019 American Community Survey. United States Department of Agriculture. Bureau of Labor Statistics.

Abbreviations: BEA, Bureau of economic analysis; CHIP, children's health insurance program; CPI, consumer price index.

**TABLE A3:** OLS regressions. Group‐specific and pooled models. Dependent variable: Log inflation adjusted hourly dentist net income

Variables	Unadjusted (1)	All races pooled (2)	White (3)	White (4)	Minoritized (5)	Asian (6)	Hispanic (7)	Black (8)
Black	−0.389***	−0.277***						
	(0.055)	(0.055)						
Asian	−0.234***	−0.109***						
	(0.022)	(0.023)						
Hispanic	−0.281***	−0.162***						
	(0.044)	(0.042)						
Other race	−0.163***	−0.132***						
	(0.043)	(0.043)						
Experience 6–10 years		0.165***	0.156***	0.154***	0.198***	0.294***	0.232	−0.145
		(0.028)	(0.031)	(0.031)	(0.061)	(0.070)	(0.175)	(0.178)
Experience 11–15 years		0.262***	0.273***	0.264***	0.234***	0.245***	0.398**	−0.009
		(0.029)	(0.031)	(0.031)	(0.067)	(0.073)	(0.187)	(0.181)
Experience 16–20 years		0.251***	0.260***	0.251***	0.232***	0.221***	0.270	0.096
		(0.027)	(0.030)	(0.030)	(0.060)	(0.068)	(0.181)	(0.174)
Experience 21–25 years		0.252***	0.243***	0.235***	0.267***	0.281***	0.391**	−0.243
		(0.027)	(0.029)	(0.029)	(0.064)	(0.072)	(0.172)	(0.197)
Experience 26–30 years		0.236***	0.230***	0.222***	0.227***	0.243***	0.487***	−0.459**
		(0.026)	(0.028)	(0.028)	(0.067)	(0.078)	(0.173)	(0.202)
Experience 31–35 years		0.228***	0.223***	0.215***	0.160**	0.111	0.527**	−0.330*
		(0.026)	(0.028)	(0.028)	(0.073)	(0.084)	(0.207)	(0.191)
Experience 36–40 years		0.144***	0.125***	0.113***	0.237***	0.237**	0.231	−0.113
		(0.028)	(0.031)	(0.031)	(0.082)	(0.105)	(0.242)	(0.180)
Experience 41+ years		−0.028	−0.035	−0.041	−0.098	−0.095	0.046	−0.504**
		(0.031)	(0.033)	(0.033)	(0.088)	(0.110)	(0.257)	(0.222)
Female		−0.214***	−0.257***	−0.265***	−0.096***	−0.070	−0.118	−0.257***
		(0.017)	(0.018)	(0.019)	(0.037)	(0.046)	(0.081)	(0.098)
Specialist		0.495***	0.496***	0.496***	0.506***	0.492***	0.494***	0.444***
		(0.011)	(0.012)	(0.012)	(0.030)	(0.039)	(0.071)	(0.093)
ZIP code <50% White population		−0.053***	−0.054***	−0.064***	−0.051	−0.039	−0.178**	0.222**
		(0.015)	(0.016)	(0.017)	(0.034)	(0.043)	(0.084)	(0.098)
Rural		0.033**	0.025	0.029*	0.162**	0.201*	0.015	0.408*
		(0.015)	(0.016)	(0.016)	(0.078)	(0.115)	(0.179)	(0.214)
Foreign trained		−0.167***	−0.363***		−0.028	0.008	−0.098	
		(0.040)	(0.060)		(0.050)	(0.068)	(0.092)	
Middle East BEA region		−0.224***	−0.210***	−0.210***	−0.384***	−0.388***	−0.335	−0.839*
		(0.025)	(0.026)	(0.026)	(0.084)	(0.099)	(0.220)	(0.472)
Great Lakes BEA region		−0.204***	−0.186***	−0.185***	−0.432***	−0.277	−0.262	−1.281***
		(0.040)	(0.042)	(0.042)	(0.131)	(0.180)	(0.344)	(0.492)
Plains BEA region		−0.216***	−0.216***	−0.214***	−0.192	−0.125	−0.104	−1.013*
		(0.042)	(0.044)	(0.044)	(0.155)	(0.221)	(0.388)	(0.557)
Southeast BEA region		−0.178***	−0.175***	−0.171***	−0.306***	−0.123	−0.191	−1.109**
		(0.037)	(0.039)	(0.039)	(0.117)	(0.165)	(0.294)	(0.484)
Southwest BEA region		−0.178***	−0.167***	−0.162***	−0.328***	−0.328*	−0.085	−1.143**
		(0.037)	(0.038)	(0.038)	(0.119)	(0.178)	(0.269)	(0.496)
Far West or/Rocky MTN		−0.166***	−0.158***	−0.165***	−0.266***	−0.267**	−0.179	−0.806*
		(0.027)	(0.028)	(0.028)	(0.083)	(0.105)	(0.212)	(0.484)
Percentage publically insured		−0.002***	−0.002***	−0.002***	−0.001**	−0.003***	0.00007	0.002
		(0.0004)	(0.0004)	(0.0004)	(0.001)	(0.001)	(0.002)	(0.002)
Regional CPI		−0.003**	−0.004***	−0.004***	−0.001	0.004	0.0004	−0.014*
		(0.001)	(0.001)	(0.001)	(0.004)	(0.006)	(0.010)	(0.007)
Observations	22,086	22,086	19,184	19,184	2902	1714	553	297
R‐squared	0.022	0.160	0.149	0.145	0.144	0.163	0.207	0.319

*Note*: SDP survey weights are applied in all estimates. Robust standard errors are in parentheses. Categorical variable for data year included in specifications (2) through (7) but not reported. ****p* < 0.01, ***p* < 0.05, **p* < 0.1. 2002–2019 American Dental Association Survey of Dental Practice.

Abbreviations: BEA, Bureau of economic analysis; CPI, consumer price index.

**TABLE A4:** OLS regressions by experience subcategory. Dependent variable: Log inflation adjusted hourly dentist net income

Variables	White, 1–20	White, 21+	Minoritized, 1–20	Minoritized, 21+	Asian, 1–20	Asian, 21+	Black, 1–20	Black, 21+	Hispanic, 1–20	Hispanic, 21+
Female	−0.240***	−0.293***	−0.0800*	−0.106*	−0.0379	−0.109	−0.441***	−0.0912	−0.0285	−0.141
	(0.024)	(0.028)	(0.047)	(0.063)	(0.056)	(0.068)	(0.136)	(0.137)	(0.107)	(0.117)
Specialist	0.501***	0.494***	0.523***	0.475***	0.462***	0.514***	0.492***	0.214	0.653***	0.320***
	(0.020)	(0.014)	(0.040)	(0.044)	(0.051)	(0.060)	(0.130)	(0.146)	(0.106)	(0.100)
ZIP code <50% White	−0.0798**	−0.0452**	−0.0196	−0.106**	0.0188	−0.134**	0.349**	0.148	−0.209*	−0.112
	(0.032)	(0.019)	(0.046)	(0.047)	(0.055)	(0.062)	(0.170)	(0.129)	(0.118)	(0.108)
Rural	0.0559**	0.00633	0.273**	0.0302	0.370**	−0.0638	0.145	0.793***	0.273	−0.204
	(0.027)	(0.019)	(0.127)	(0.082)	(0.172)	(0.131)	(0.424)	(0.177)	(0.240)	(0.219)
Foreign trained	−0.259***	−0.416***	−0.122	0.0246	0.00292	0.0231			−0.254	−0.104
	(0.094)	(0.076)	(0.087)	(0.058)	(0.121)	(0.082)			(0.162)	(0.111)
Middle East BEA region	−0.186***	−0.221***	−0.356***	−0.444**	−0.374***	−0.306	−0.365	−1.853***	−0.337	−0.473
	(0.053)	(0.029)	(0.010)	(0.189)	(0.121)	(0.191)	(0.301)	(0.273)	(0.299)	(0.288)
Great Lakes BEA region	−0.0964	−0.236***	−0.252	−0.718***	−0.0937	−0.620**	−0.787**	−2.212***	0.339	−0.971**
	(0.080)	(0.047)	(0.167)	(0.236)	(0.234)	(0.305)	(0.350)	(0.354)	(0.506)	(0.454)
Plains BEA region	−0.149*	−0.263***	0.0155	−0.450*	0.00505	−0.219	−0.271	−2.372***	0.204	−0.608
	(0.084)	(0.050)	(0.195)	(0.262)	(0.298)	(0.353)	(0.514)	(0.620)	(0.541)	(0.504)
Southeast BEA region	−0.146**	−0.188***	−0.200	−0.464**	−0.0463	−0.144	−0.499	−2.141***	0.128	−0.505
	(0.074)	(0.043)	(0.147)	(0.217)	(0.209)	(0.303)	(0.336)	(0.318)	(0.424)	(0.370)
Southwest BEA region	−0.0814	−0.221***	−0.281*	−0.380*	−0.359	−0.0378	−0.368	−2.424***	0.274	−0.520
	(0.072)	(0.043)	(0.151)	(0.213)	(0.225)	(0.300)	(0.386)	(0.364)	(0.372)	(0.354)
Far West/Rocky MTN	−0.116**	−0.180***	−0.222**	−0.325*	−0.267**	−0.169	−0.372	−1.833***	0.208	−0.542**
	(0.055)	(0.032)	(0.099)	(0.185)	(0.134)	(0.198)	(0.316)	(0.309)	(0.300)	(0.267)
Percent public insured	−0.0004	−0.003***	−0.0009	−0.002**	−0.002	−0.005***	−0.0004	0.006**	0.0003	−0.0006
	(0.0006)	(0.0006)	(0.0009)	(0.001)	(0.001)	(0.001)	(0.002)	(0.002)	(0.003)	(0.002)
Regional CPI	−0.003	−0.004***	0.002	−0.004	0.008	−0.004	−0.008	−0.013	0.011	−0.004
	(0.003)	(0.002)	(0.005)	(0.006)	(0.008)	(0.009)	(0.012)	(0.010)	(0.015)	(0.012)
Experience 6–10 years	0.164***		0.205***		0.318***		−0.179		0.181	
	(0.0307)		(0.0614)		(0.0694)		(0.221)		(0.171)	
Experience 11–15 years	0.283***		0.247***		0.267***		0.0293		0.391**	
	(0.0312)		(0.0684)		(0.0746)		(0.199)		(0.189)	
Experience 16–20 years	0.274***		0.241***		0.232***		0.0592		0.234	
	(0.0303)		(0.0610)		(0.0686)		(0.201)		(0.187)	
Experience 26–30 years		−0.0140		−0.0450		−0.0516		−0.0643		0.0734
		(0.021)		(0.0605)		(0.0806)		(0.165)		(0.114)
Experience 31–35 years		−0.0221		−0.117*		−0.172*		0.0149		0.0976
		(0.021)		(0.0689)		(0.0901)		(0.171)		(0.138)
Experience 36–40 years		−0.121***		−0.0298		−0.0266		0.345**		−0.216
		(0.024)		(0.074)		(0.101)		(0.169)		(0.197)
Experience 41+ years		−0.279***		−0.338***		−0.328***		−0.0503		−0.145
		(0.027)		(0.085)		(0.112)		(0.223)		(0.202)
Observations	5892	13,292	1509	1393	948	766	144	153	260	293
R‐squared	0.177	0.137	0.154	0.163	0.166	0.218	0.416	0.445	0.346	0.227

*Note*: SDP survey weights are applied in all estimates. Indicator for foreign trained dentist is excluded from the model for Black dentists due to the small sample cell size. Robust standard errors are in parentheses. Categorical variable for data year included in all specifications but not reported. ****p* < 0.01, ***p* < 0.05, **p* < 0.1. 2002–2019 American Dental Association Survey of Dental Practice.

Abbreviations: BEA, Bureau of economic analysis; CPI, consumer price index.

**TABLE A5:** Linear Blinder‐Oaxaca decomposition, White reference coefficients

Variables	White versus Minoritized		White versus Asian		White versus Hispanic		White versus Black	
	Explained	Unexplained	Explained	Unexplained	Explained	Unexplained	Explained	Unexplained
Experience	−0.007**	0.003	−0.003	−0.016	−0.014***	−0.124	−0.008	0.339**
	(0.004)	(0.051)	(0.004)	(0.052)	(0.004)	(0.143)	(0.006)	(0.140)
Gender	0.050***	−0.055***	0.053***	−0.066***	0.050***	−0.047*	0.066***	−0.003
	(0.004)	(0.014)	(0.005)	(0.017)	(0.006)	(0.029)	(0.009)	(0.040)
Specialty	0.010***	−0.002	0.011**	0.0006	0.005	0.0003	0.019*	0.008
	(0.004)	(0.005)	(0.005)	(0.007)	(0.008)	(0.013)	(0.010)	(0.014)
Race and ethnicity of Zip code	0.020***	−0.001	0.023***	−0.009	0.019***	0.067	0.026***	−0.167***
	(0.006)	(0.021)	(0.007)	(0.028)	(0.006)	(0.046)	(0.007)	(0.059)
Urbanicity	0.002	−0.005*	0.003	−0.005	0.002	0.0004	0.003*	−0.009
	(0.002)	(0.003)	(0.002)	(0.003)	(0.002)	(0.006)	(0.002)	(0.006)
School (foreign or domestic)	0.046***	−0.047***	0.042***	−0.049***	0.090***	−0.070**		
	(0.008)	(0.011)	(0.008)	(0.012)	(0.017)	(0.030)		
Year	−0.008*	0.163	−0.008**	0.420	−0.008	0.111	−0.006*	−0.717**
	(0.004)	(0.202)	(0.004)	(0.307)	(0.007)	(0.474)	(0.004)	(0.362)
BEA region	−0.004	0.134	−0.007	0.101	−0.002	0.017	0.0010**	0.826*
	(0.005)	(0.091)	(0.008)	(0.111)	(0.004)	(0.236)	(0.004)	(0.470)
Percentage with Medicaid/CHIP	0.017***	−0.006	0.018***	0.018	0.011***	−0.024	0.027***	−0.077**
	(0.004)	(0.013)	(0.004)	(0.016)	(0.003)	(0.023)	(0.007)	(0.036)
Regional CPI	0.019***	−0.582	0.024***	−1.721	0.020**	−0.938	−0.007	2.070
	(0.007)	(0.917)	(0.009)	(1.348)	(0.008)	(2.270)	(0.006)	(1.515)
Constant		0.508		1.403		1.114		−2.011
		(0.800)		(1.155)		(2.001)		(1.478)
Unadjusted log earnings gap	0.254***		0.234***		0.281***		0.389***	
	(0.0176)	%	(0.0212)	%	(0.0409)	%	(0.0491)	%
Total explained by model	0.145***	57.1%	0.155***	66.2%	0.175***	62.3%	0.131***	33.7%
	(0.0127)		(0.0140)		(0.0215)		(0.0188)	
Unexplained log earnings gap	0.109***	42.9%	0.0786***	33.8%	0.106**	37.7%	0.258***	66.3%
	(0.0210)		(0.0243)		(0.0434)		(0.0499)	
Number of observations	22,086		20,898		19,737		19,481	
White observations	19,184		19,184		19,184		19,184	
Minority observations	2902		1714		553		297	

*Note*: SDP survey weights applied in all estimates. The indicator for foreign trained dentist is excluded from the model for Black dentists due to the small sample cell size. Robust standard errors are in parentheses. Reference coefficients based on coefficients from all‐White sample. ****p* < 0.01, ***p* < 0.05, **p* < 0.1. 2002–2019 American Dental Association Survey of Dental Practice.

Abbreviations: BEA, Bureau of economic analysis; CHIP, children's health insurance program; CPI, consumer price index.

**TABLE A6:** Linear Blinder‐Oaxaca decomposition (Unweighted)

Variables	White versus Minoritized		White versus Asian		White versus Hispanic		White versus Black	
	Explained	Unexplained	Explained	Unexplained	Explained	Unexplained	Explained	Unexplained
Experience	−0.011***	0.060	−0.007**	0.061	−0.018***	−0.072	−0.009	0.177
	(0.003)	(0.041)	(0.004)	(0.048)	(0.005)	(0.105)	(0.006)	(0.115)
Gender	0.043***	−0.030***	0.047***	−0.039***	0.046***	−0.030*	0.059***	−0.011
	(0.003)	(0.008)	(0.004)	(0.011)	(0.006)	(0.018)	(0.008)	(0.027)
Specialty	0.004	−0.002	0.007	−0.000	−0.011	0.011	0.018	0.003
	(0.005)	(0.010)	(0.006)	(0.013)	(0.010)	(0.023)	(0.013)	(0.025)
Race and ethnicity of ZIP code	0.018***	0.014	0.020***	0.006	0.014***	0.032	0.019***	−0.053
	(0.004)	(0.013)	(0.005)	(0.020)	(0.004)	(0.026)	(0.005)	(0.039)
Urbanicity	0.002	−0.004	0.002	−0.005*	0.001	0.007	0.001	−0.017**
	(0.001)	(0.003)	(0.001)	(0.003)	(0.001)	(0.006)	(0.001)	(0.008)
School (foreign or domestic)	0.015***	−0.014***	0.016***	−0.022***	0.041***	−0.011		
	(0.003)	(0.004)	(0.004)	(0.006)	(0.008)	(0.013)		
Year	−0.008***	0.149	−0.006**	0.271	−0.010*	−0.103	−0.009*	−0.200
	(0.003)	(0.141)	(0.003)	(0.208)	(0.005)	(0.299)	(0.005)	(0.335)
BEA region	−0.005	0.038	−0.005	0.001	−0.002	−0.009	0.009***	0.846***
	(0.004)	(0.074)	(0.006)	(0.091)	(0.003)	(0.172)	(0.003)	(0.261)
Percentage with Medicaid/CHIP	0.010***	0.011	0.010***	0.024**	0.007***	−0.002	0.020***	−0.015
	(0.002)	(0.007)	(0.002)	(0.010)	(0.002)	(0.013)	(0.005)	(0.028)
Regional CPI	0.018***	−0.525	0.024***	−0.756	0.015***	−0.203	−0.0005	0.418
	(0.005)	(0.624)	(0.007)	(0.934)	(0.006)	(1.372)	(0.006)	(1.291)
Constant		0.450		0.577		0.507		−0.879
		(0.555)		(0.815)		(1.214)		(1.188)
Unadjusted log earnings gap	0.232***		0.223***		0.211***		0.378***	
	(0.014)	%	(0.018)	%	(0.030)	%	(0.039)	%
Total explained by model	0.086***	37.1%	0.106***	47.5%	0.083***	39.3%	0.108***	28.6%
	(0.009)		(0.011)		(0.016)		(0.018)	
Unexplained log earnings gap	0.146***	62.9%	0.117***	52.5%	0.127***	60.7%	0.270***	71.4%
	(0.014)		(0.018)		(0.028)		(0.036)	
Number of observations	22,086		20,898		19,737		19,481	
White observations	19,184		19,184		19,184		19,184	
Minority observations	2902		1714		553		297	

*Note*: Indicator for foreign trained dentist is excluded fromn the model for Black dentists due to the small sample cell size. Robust standard errors are in parentheses. Reference coefficients based on the pooled regression model. ****p* < 0.01, ***p* < 0.05, **p* < 0.1. 2002–2019 American Dental Association Survey of Dental Practice.

Abbreviations: BEA, Bureau of economic analysis; CHIP, children's health insurance program; CPI, consumer price index.

**TABLE A7:** Linear Blinder‐Oaxaca decomposition (2015–2018)

Variables	White versus Minoritized	
	Explained	Unexplained
Experience	−0.028***	0.013
	(0.007)	(0.117)
Gender	0.045***	−0.035
	(0.007)	(0.033)
Specialty	0.015*	0.006
	(0.008)	(0.018)
Race and ethnicity of ZIP code	0.024**	−0.019
	(0.010)	(0.040)
Urbanicity	0.005	−0.003
	(0.003)	(0.005)
School (foreign or domestic)	0.009	−0.030**
	(0.008)	(0.012)
Year	0.004	−0.083
	(0.003)	(0.103)
BEA region	−0.006	0.287
	(0.012)	(0.221)
Percentage with Medicaid/CHIP	0.031***	0.004
	(0.007)	(0.025)
Regional CPI	0.018**	0.657
	(0.008)	(2.013)
Constant		−0.688
		(2.089)
Unadjusted log earnings gap	0.224***	
	(0.044)	%
Total explained by model	0.115***	51.3%
	(0.019)	
Unexplained log earnings gap	0.109**	48.7%
	(0.048)	
Number of observations	4808	
White observations	4077	
Minority observations	731	

*Note*: SDP survey weights applied in all estimates. Robust standard errors are in parentheses. Reference coefficients based on pooled regression model. ****p* < 0.01, ***p* < 0.05, **p* < 0.1. 2016–2019 American Dental Association Survey of Dental Practice.

Abbreviations: BEA, Bureau of economic analysis; CHIP, children's health insurance program; CPI, consumer price index.

**TABLE A8:** Linear Blinder‐Oaxaca decomposition by experience subcategory

Variables	White versus Asian, Experience ≤20 Years		White versus Asian, Experience >20 Years		White versus Hispanic, Experience ≤20 Years		White versus Hispanic, Experience >20 Years		White versus Black, Experience ≤20 Years		White versus Black, Experience >20 Years	
	Explained	Unexplained	Explained	Unexplained	Explained	Unexplained	Explained	Unexplained	Explained	Unexplained	Explained	Unexplained
Experience	0.007*	‐0.026	‐0.019***	0.028	‐0.002	‐0.020	‐0.027***	‐0.051	‐0.003	0.234	‐0.010	‐0.090
	(0.004)	(0.059)	(0.004)	(0.053)	(0.007)	(0.152)	(0.005)	(0.082)	(0.008)	(0.199)	(0.007)	(0.125)
Gender	0.032***	‐0.079***	0.041***	‐0.038**	0.032***	‐0.083*	0.054***	‐0.037	0.063***	0.102	0.050***	‐0.051
	(0.005)	(0.029)	(0.006)	(0.018)	(0.008)	(0.048)	(0.009)	(0.036)	(0.012)	(0.089)	(0.012)	(0.042)
Specialty	0.016**	0.006	0.005	‐0.003	0.010	‐0.026	0.001	0.031	0.009	0.001	0.035***	0.031
	(0.006)	(0.011)	(0.007)	(0.013)	(0.012)	(0.021)	(0.011)	(0.021)	(0.016)	(0.029)	(0.013)	(0.022)
Race and ethnicity of ZIP code	0.024**	‐0.047	0.028***	0.057	0.037***	0.063	0.016**	0.036	0.022**	‐0.208**	0.027***	‐0.142
	(0.011)	(0.034)	(0.009)	(0.051)	(0.012)	(0.069)	(0.007)	(0.067)	(0.011)	(0.106)	(0.010)	(0.118)
Urbanicity	0.007***	‐0.009	0.001	0.002	0.005**	‐0.008	0.000	0.007	0.007**	‐0.002	0.001	‐0.026*
	(0.003)	(0.006)	(0.002)	(0.005)	(0.003)	(0.010)	(0.002)	(0.009)	(0.003)	(0.010)	(0.002)	(0.014)
School (foreign or domestic)	0.006*	‐0.010	0.054***	‐0.067***	0.047***	‐0.001	0.100***	‐0.071*				
	(0.003)	(0.007)	(0.015)	(0.023)	(0.016)	(0.032)	(0.024)	(0.043)				
Year	‐0.001	0.536	‐0.011	0.132	‐0.003	0.657	‐0.012	‐0.369	‐0.002	‐0.758	‐0.012*	0.568
	(0.005)	(0.501)	(0.009)	(0.530)	(0.006)	(0.714)	(0.014)	(0.732)	(0.007)	(0.701)	(0.007)	(0.605)
BEA region	0.008	0.109	‐0.012	0.008	0.00009	‐0.263	‐0.001	0.357	0.011	0.323	0.005	1.812***
	(0.011)	(0.181)	(0.012)	(0.234)	(0.006)	(0.374)	(0.005)	(0.357)	(0.008)	(0.387)	(0.006)	(0.370)
Percentage with Medicaid/CHIP	0.008	0.018	0.030***	0.015	0.001	‐0.009	0.019***	‐0.030	0.006	‐0.004	0.042***	‐0.173***
	(0.005)	(0.022)	(0.005)	(0.021)	(0.003)	(0.038)	(0.005)	(0.026)	(0.007)	(0.051)	(0.011)	(0.059)
Regional CPI	0.005‐2.470	0.045***	‐0.123	0.009‐3.164	0.038***	‐0.116	‐0.004	0.979	‐0.012	1.703		
	(0.013)	(2.189)	(0.016)	(2.464)	(0.009)	(3.727)	(0.015)	(3.089)	(0.008)	(3.245)	(0.011)	(2.851)
Constant		2.077		0.059		3.030		0.280		‐0.461		‐3.287
		(1.899)		(2.106)		(3.333)		(2.701)		(2.989)		(2.600)
Unadjusted log earnings gap	0.220***		0.231***		0.314***		0.227***		0.315***		0.471***	
	(0.033)	%	(0.037)	%	(0.065)	%	(0.060)	%	(0.085)	%	(0.074)	%
Total explained by model	0.114***	51.8%	0.162***	70.1%	0.139***	44.3%	0.189***	83.3%	0.108***	34.3%	0.126***	26.8%
	(0.017)		(0.024)		(0.026)		(0.031)		(0.028)		(0.026)	
Unexplained log earnings gap	0.106***	48.2%	0.069*	29.9%	0.175***	55.7%	0.038	16.7%	0.207**	65.7%	0.345***	73.2%
	(0.035)		(0.039)		(0.063)		(0.063)		(0.084)		(0.075)	
Total observations	6840		14,058		6152		13,585		6036		13,445	
White observations	5892		13,292		5892		13,292		5892		13,292	
Minority observations	948		766		260		293		144		153	

*Note*: SDP survey weights applied in all estimates. Indicator for foreign trained dentist is excluded from the model for Black dentists due to the small sample cell size. Robust standard errors are in parentheses. Reference coefficients based on the pooled regression model. ****p* < 0.01, ***p* < 0.05, **p* < 0.1. 2002‐2019 American Dental Association Survey of Dental Practice.

Abbreviations: BEA, Bureau of economic analysis; CHIP, children's health insurance program; CPI, consumer price index.

**TABLE A9:** White versus Asian RIF Blinder‐Oaxaca decomposition (Unweighted)

Variables	RIF 10th percentile		RIF 50th percentile		RIF 90th percentile	
	Explained	Unexplained	explained	unexplained	explained	unexplained
Experience	−0.025***	0.004	0.001	0.067	−0.008	−0.040
	(0.006)	(0.124)	(0.004)	(0.065)	(0.005)	(0.071)
Gender	0.071***	−0.116***	0.050***	−0.025*	0.034***	−0.027
	(0.008)	(0.024)	(0.005)	(0.015)	(0.005)	(0.020)
Specialty	0.005	0.003	0.007	−0.015	0.008	−0.011
	(0.004)	(0.021)	(0.006)	(0.016)	(0.007)	(0.027)
Race and ethnicity of ZIP code	0.027**	0.032	0.030***	−0.026	−0.006	0.053
	(0.011)	(0.040)	(0.006)	(0.028)	(0.009)	(0.039)
Urbanicity	0.008***	−0.002	0.003	−0.004	−0.006***	−0.012**
	(0.003)	(0.005)	(0.002)	(0.003)	(0.002)	(0.006)
School (foreign or domestic)	0.042***	−0.047**	0.022***	−0.033***	0.019**	−0.018
	(0.013)	(0.020)	(0.006)	(0.011)	(0.008)	(0.015)
Year	−0.008*	0.112	−0.008**	0.156	−0.003	0.912***
	(0.005)	(0.414)	(0.003)	(0.245)	(0.003)	(0.347)
BEA region	0.012	0.070	−0.019**	0.138	0.009	−0.272
	(0.013)	(0.145)	(0.008)	(0.126)	(0.010)	(0.179)
Percentage with Medicaid/CHIP	0.025***	0.011	0.009***	0.031**	−0.005*	0.034**
	(0.005)	(0.023)	(0.002)	(0.013)	(0.003)	(0.015)
Regional CPI	0.025*	−0.184	0.032***	0.220	0.006	−2.586*
	(0.013)	(1.995)	(0.009)	(1.164)	(0.010)	(1.465)
Constant		0.188		−0.400		2.095
		(1.733)		(1.013)		(1.291)
Unadjusted log earnings gap	0.252***		0.236***		0.176***	
	(0.031)		(0.022)		(0.030)	
Total explained by model	0.181***	71.8%	0.127***	53.8%	0.047***	26.7%
	(0.022)		(0.013)		(0.016)	
Unexplained log earnings gap	0.071*	28.2%	0.109***	46.2%	0.129***	73.3%
	(0.038)		(0.023)		(0.032)	
Number of observations	20,898		20,898		20,898	
White observations	19,184		19,184		19,184	
Asian observations	1714		1714		1714	

*Note*: RIF‐Re‐centered influence function. Robust standard errors are in parentheses. Reference coefficients based on all‐White sample. ****p* < 0.01, ***p* < 0.05, **p* < 0.1. 2002–2019 American Dental Association Survey of Dental Practice.

Abbreviations: BEA, Bureau of economic analysis; CHIP, children's health insurance program; CPI, consumer price index.

**TABLE A10:** White versus Hispanic RIF Blinder‐Oaxaca decomposition (Unweighted)

Variables	RIF 10th Percentile		RIF 50th Percentile		RIF 90th Percentile	
	explained	Unexplained	Explained	Unexplained	Explained	Unexplained
Experience	−0.032***	0.013	−0.012**	−0.044	−0.017***	−0.003
	(0.006)	(0.246)	(0.005)	(0.131)	(0.005)	(0.145)
Gender	0.066***	−0.030	0.047***	−0.044*	0.031***	0.005
	(0.010)	(0.041)	(0.006)	(0.025)	(0.005)	(0.028)
Specialty	−0.008	−0.065	−0.011	0.019	−0.014	0.104**
	(0.007)	(0.044)	(0.010)	(0.034)	(0.013)	(0.041)
Race and ethnicity of ZIP Code	0.020**	0.065	0.022***	0.003	−0.004	0.082*
	(0.008)	(0.059)	(0.005)	(0.039)	(0.006)	(0.042)
Urbanicity	0.006***	0.001	0.002	0.008	−0.005***	0.012
	(0.002)	(0.012)	(0.002)	(0.009)	(0.002)	(0.008)
School (foreign or domestic)	0.062***	−0.006	0.033***	−0.004	0.029**	0.019
	(0.020)	(0.037)	(0.010)	(0.023)	(0.012)	(0.024)
Year	−0.016*	0.410	−0.010*	−0.186	0.000	−0.392
	(0.009)	(0.521)	(0.006)	(0.445)	(0.007)	(0.523)
BEA region	0.002	0.167	−0.007**	0.360	0.003	−0.315
	(0.005)	(0.228)	(0.004)	(0.249)	(0.004)	(0.304)
Percentage with Medicaid/CHIP	0.022***	−0.044	0.008***	0.013	−0.005	0.005
	(0.005)	(0.032)	(0.002)	(0.021)	(0.003)	(0.023)
Regional CPI	0.016*	−0.419	0.020***	0.287	0.004	1.037
	(0.009)	(2.304)	(0.008)	(1.925)	(0.006)	(2.730)
Constant		0.017		−0.304		−0.375
		(2.045)		(1.701)		(2.454)
Unadjusted log earnings gap	0.247***		0.197***		0.201***	
	(0.058)	%	(0.039)	%	(0.046)	%
Total explained by model	0.139***	56.3%	0.090***	45.7%	0.022	10.9%
	(0.027)		(0.018)		(0.020)	
Unexplained log earnings gap	0.109*	43.7%	0.107***	54.3%	0.179***	89.1%
	(0.062)		(0.039)		(0.047)	
Number of observations	19,737		19,737		19,737	
White observations	19,184		19,184		19,184	
Hispanic observations	553		553		553	

*Note*: RIF‐Re‐centered influence function. Robust standard errors are in parentheses. Reference coefficients based on all‐White sample. ****p* < 0.01, ***p* < 0.05, **p* < 0.1. 2002–2019 American Dental Association Survey of Dental Practice.

Abbreviations: BEA, Bureau of economic analysis; CHIP, children's health insurance program; CPI, consumer price index.

**TABLE A11:** White versus Black RIF Blinder‐Oaxaca decomposition (Unweighted)

Variables	RIF 10th percentile		RIF 50th percentile		RIF 90th percentile	
	Explained	Unexplained	Explained	Unexplained	Explained	Unexplained
Experience	−0.020***	0.474***	−0.004	0.189	−0.007	−0.480**
	(0.007)	(0.136)	(0.006)	(0.205)	(0.006)	(0.243)
Gender	0.082***	−0.055	0.058***	−0.026	0.039***	0.009
	(0.013)	(0.048)	(0.008)	(0.039)	(0.007)	(0.060)
Specialty	0.013	0.006	0.019	0.078**	0.024	−0.062
	(0.010)	(0.032)	(0.013)	(0.037)	(0.017)	(0.073)
Race and ethnicity of ZIP code	0.029***	−0.111*	0.031***	−0.096*	−0.005	0.033
	(0.011)	(0.065)	(0.007)	(0.058)	(0.009)	(0.097)
Urbanicity	0.007***	−0.011	0.002	−0.022**	−0.005***	−0.015
	(0.003)	(0.008)	(0.002)	(0.011)	(0.002)	(0.022)
Year	−0.012	0.235	−0.011*	−0.333	−0.002	0.375
	(0.008)	(0.437)	(0.006)	(0.496)	(0.005)	(0.944)
BEA region	0.011**	0.416*	0.007*	0.620**	0.013***	2.366***
	(0.006)	(0.244)	(0.004)	(0.286)	(0.005)	(0.765)
Percentage with Medicaid/CHIP	0.063***	−0.098**	0.022***	−0.051	−0.012	0.068
	(0.013)	(0.047)	(0.006)	(0.038)	(0.008)	(0.075)
Regional CPI	−0.000	0.216	−0.001	0.873	−0.000	−0.886
	(0.006)	(2.169)	(0.008)	(1.866)	(0.001)	(3.114)
Constant		−0.920		−0.968		−1.046
		(1.934)		(1.732)		(2.866)
Unadjusted log earnings gap	0.323***		0.387***		0.408***	
	(0.056)	%	(0.050)	%	(0.087)	%
Total explained by model	0.173***	53.6%	0.123***	31.8%	0.045**	11.0%
	(0.025)		(0.020)		(0.023)	
Unexplained log earnings gap	0.150**	46.4%	0.263***	68.2%	0.363***	89.0%
	(0.059)		(0.050)		(0.084)	
Number of observations	19,481		19,481		19,481	
White observations	19,184		19,184		19,184	
Black observations	297		297		297	

*Note*: Indicator for foreign trained dentist is excluded from the model for Black dentists due to the small sample cell size. RIF‐Re‐centered influence function. Robust standard errors are in parentheses. Reference coefficients based on all‐White sample. ****p* < 0.01, ***p* < 0.05, **p* < 0.1. 2002–2019 American Dental Association Survey of Dental Practice.

Abbreviations: BEA, Bureau of economic analysis; CHIP, children's health insurance program.
