# Supplementary material for: A closer look at disparities in earnings between white and minoritized dentists
Source: Health Serv Res. 2022 Nov 14;58(3):705–32. doi: 10.1111/1475-6773.14095 (PMC10154170; doi:10.1111/1475-6773.14095)
Supplement: Supplementary file 1 — Appendix S1. Supporting Information. [file HESR-58-705-s001.docx]

**Technical Appendix**

**Linear Blinder-Oaxaca Decomposition**

We employed the commonly used Blinder^[[1]](#endnote-1)^ and Oaxaca^[[2]](#endnote-2)^ methodology to decompose differences in inflation-adjusted hourly net income between racial/ethnic subgroups. In our analysis, we separately compared White Dentists to minoritized dentists, White dentists to Asian dentists, White dentists to Hispanic dentists, and White dentists to Black dentists. The Blinder-Oaxaca methodology begins with a simple linear earnings model that is separable in observable and unobservable characteristics

$$Y_{g}=\boldsymbol{X}\beta_{g}+v_{g} g=white,Minority (1)$$

where *Minority* is defined as Asian, Black, or Hispanic. The dependent variable, $Y_{g},$ represents log of hourly inflation-adjusted net income of the two groups we examined, Whites and minoritized dentists. $\boldsymbol{X}$ is a vector of observables that includes female gender, a categorical variable for experience in five-year increments (1-5, 6-10,10-15, 16-20, 21-25, 26-30, 31-35, 36-40, 41 or more years), a binary variable identifying whether a dentist graduated from a foreign dental school, a binary indicator identifying a rural dentist, a binary variable identifying whether a dentist has a practice in a majority non-White ZIP code, a binary variable for a non-general practice (GP) or specialist dentist (pediatric dentist, endodontist, periodontist, prosthodontist, orthodontist, oral surgeon, or other specialist), a categorical variable for BEA region (New England, Mideast, Great Lakes, Plains, Southeast, Southwest, Rocky Mountain/Far West), a categorical variable for data year, a regional consumer price index (CPI) that varies by census region and year, and the percentage of a dentist’s patients that are publically insured. We included a binary foreign dental school indicator variable in our specification since previous research concluded that country of origin and location of study explain the level of a worker’s earnings^[[3]](#endnote-3)^. We also assumed that the unobservable error term, $v_{g}$, has a zero conditional mean, E($v_{g}\left| \boldsymbol{X} \right)$=0.

For each racial/ethnic group, we estimated separate ordinary least squares regressions (OLS) to estimate $\hat{\beta}_{g}$. We used these parameter estimates to decompose the mean differences in the outcome variable between the two groups, $\bar{Y}_{M}-\bar{Y}_{W}$, into an “explained” and “unexplained” component, where $\bar{Y}_{M}$ is the mean outcome for the minority group and $\bar{Y}_{w}$ is the mean outcome for Whites. In the labor economics literature examining differences in earnings between groups, the “explained” component is also referred to as the “composition” effect while the “unexplained” component is referred to as the “wage structure” effect or the effect due to discrimination.^[[4]](#endnote-4)^ By estimating parameter estimates, $\hat{\beta}_{g}$, for each group and computing group specific averages for the observed covariates, $\bar{X}_{W}$ and $\bar{X}_{M}$, the two-fold decomposition can be written as

$\bar{Y}_{M}-\bar{Y}_{W}=(\bar{X}_{M}$- $\bar{X}_{W}$) $\hat{\beta}_{W}$+$\bar{X}_{M}$($\hat{\beta}_{M}-\hat{\beta}_{W})$ (2)

where the first term on the right hand side of equation (2) is the “explained” component and second term is the “unexplained” component. The “explained” component captures the differential in earnings that is due to observable average differences between minorities (Asian, Hispanic and Black) and Whites. For example, if specialists typically earn more than GP dentists and a higher proportion of Whites are specialists, this could explain a portion of the observable gap in earnings between White and minoritized dentists. The “unexplained” component captures differences in the relationship between a given characteristic, such as experience and earnings, for minoritized dentists and White dentists. For example, if White dentists have a greater return to experience than Asian dentists, this would be captured in the “unexplained” component of the Blinder-Oaxaca decomposition. The decomposition in equation (2) is written in terms of “White” reference coefficients. However, there may be no rationale to writing the decomposition in terms of either group. Hence, in our main results, we present a decomposition based on reference coefficients from a pooled regression model as done in previous research, but with a group indicator (e.g., White/minority) variable added on as an additional covariate in the pooled model.^[[5]](#endnote-5)^ ^[[6]](#endnote-6)^ ^[[7]](#endnote-7)^ In our results, the total “explained” and total “unexplained” component of the decomposition are presented as a percentage of the total difference in earnings between minoritized and White dentists.

**Oaxaca Decomposition with Re-Centered Influence Functions**

Given that dentist earnings vary within subgroups, we were also interested in the effect of observables on specific quantiles of a distribution. Firpo, Fortin and Lemieux^[[8]](#endnote-8)^ devised a methodology to evaluate the effect of discrete changes in observables on specific unconditional quantiles of interest. Using a re-centered influence function, defined as $RIF\left( Y;Q_{\tau};F_{Y} \right),$ one can perform a Blinder-Oaxaca style decomposition for other statistics besides the mean such as a quantile, where $Y$ is the outcome variable, log of inflation-adjusted hourly net income, $Q_{\tau}$ is a specific quantile, and $F_{Y}$is the cumulative distribution function (CDF) of the outcome variable. Conditional on observables, E($RIF\left( Y;Q_{\tau};F_{Y} \right)\left| \boldsymbol{X} \right),$ one can estimate an unconditional quantile regression model based on the RIF. For specific quantiles, the RIF is defined as

$$RIF\left( Y;Q_{\tau};F_{Y} \right)=Q_{\tau}+\frac{\tau-\mathbb{1\{}Y\leq Q_{\tau}\}}{f_{Y}(Q_{\tau})}$$

The density, $f_{Y}\left( Q_{\tau} \right),$is estimated via kernel density methods and $\mathbb{1}\left\{ \cdot\right\}$ is an indicator function equal to 1 if the outcome variable is at or below the quantile of interest. By estimating E($RIF\left( Y;Q_{\tau};F_{Y} \right)\left| \boldsymbol{X} \right)$ separately for Whites and minorities using OLS, we can then apply the Blinder-Oaxaca decomposition for specific earnings quantiles.^4^ ^[[9]](#endnote-9)^ In our analysis, using the RIF as the dependent variable, we conducted a Blinder-Oaxaca decomposition to examine the White versus minoritized dentist earnings gap for the 10^th^ percentile, 50^th^ percentile, and 90^th^ percentile.

1. Blinder AS. Wage discrimination: reduced form and structural estimates. *J Hum Resour.* 1973; 8(4):436–455. [↑](#endnote-ref-1)
2. Oaxaca R. Male-female wage differentials in urban labor markets. *Int Econ Rev.* 1973; 14(3):693–709. [↑](#endnote-ref-2)
3. Fortin N, Lemieux T, Torres J. Foreign human capital and the earnings gap between immigrants and Canadian-born workers. *Labour Econ.* 2016; 41:104-119. [↑](#endnote-ref-3)
4. Fortin N, Lemieux T, Firpo S. Decomposition methods in economics. In: Card D, Ashenfelter O., eds. *Handbook of Labor Economics*, 4th ed. Amsterdam: Elsevier; 2011:1-102. [↑](#endnote-ref-4)
5. Neumark D. Employers’ discriminatory behavior and the estimation of wage discrimination. *J Hum Resour*. 1988; 23(3): 279–295. [↑](#endnote-ref-5)
6. Jann B. The Blinder-Oaxaca decomposition for linear regression models. *The Stata Journal*. 2008;8(4): 453-479. [↑](#endnote-ref-6)
7. Elder TE, Goddeeris JH, Haider SJ. Unexplained gaps and Oaxaca-Blidner decompositions. *Labour Econ.* 2010;17: 284-290. [↑](#endnote-ref-7)
8. Firpo SP, Fortin NM, Lemieux T. Unconditional quantile regressions. *Econometrica*. 2009; 77(3): 953-973. [↑](#endnote-ref-8)
9. Firpo SP, Fortin NM, Lemieux T. Decomposing wage distributions using recentered influence functions. *Econometrics.* 2018;6(28):1-40. [↑](#endnote-ref-9)
